# Finite element analysis of bone remodelling with piezoelectric effects using an open-source framework

**DOI:** 10.1007/s10237-021-01439-3

**Published:** 2021-03-19

**Authors:** Yogesh Deepak Bansod, Maeruan Kebbach, Daniel Kluess, Rainer Bader, Ursula van Rienen

**Affiliations:** 1 Institute of General Electrical Engineering, University of Rostock, 18051 Rostock, Germany; 2 Department of Orthopaedics, University Medicine Rostock, 18057 Rostock, Germany; 3 Department Life, Light & Matter, University of Rostock, 18051 Rostock, Germany; 4 Department Ageing of Individuals and Society, University of Rostock, 18051 Rostock, Germany

**Keywords:** Bone remodelling, Piezoelectricity, Electrical stimulation, Open-source, Finite element modelling, Hounsfield units (HU)

## Abstract

Bone tissue exhibits piezoelectric properties and thus is capable of transforming mechanical stress into electrical potential. Piezoelectricity has been shown to play a vital role in bone adaptation and remodelling processes. Therefore, to better understand the interplay between mechanical and electrical stimulation during these processes, strain-adaptive bone remodelling models without and with considering the piezoelectric effect were simulated using the Python-based open-source software framework. To discretise numerical attributes, the finite element method (FEM) was used for the spatial variables and an explicit Euler scheme for the temporal derivatives. The predicted bone apparent density distributions were qualitatively and quantitatively evaluated against the radiographic scan of a human proximal femur and the bone apparent density calculated using a bone mineral density (BMD) calibration phantom, respectively. Additionally, the effect of the initial bone density on the resulting predicted density distribution was investigated globally and locally. The simulation results showed that the electrically stimulated bone surface enhanced bone deposition and these are in good agreement with previous findings from the literature. Moreover, mechanical stimuli due to daily physical activities could be supported by therapeutic electrical stimulation to reduce bone loss in case of physical impairment or osteoporosis. The bone remodelling algorithm implemented using an open-source software framework facilitates easy accessibility and reproducibility of finite element analysis made.

## Introduction

Bone is the main constituent of the human musculoskeletal system that provides structural integrity to the body, protects the internal organs, acts as a store for minerals and lipids, provides muscle attachment sites and facilitates body movements (Cowin 2001). It is a living tissue that adapts its morphology to the mechanical loading environment to which it is exposed and this adaptation process is called bone remodelling, a process of bone formation and resorption (Wolff 1893; Robling and Turner 2009). Since this process has a significant effect on the overall health of the entire body, bone remodelling studies are of great interest. Moreover, the piezoelectric properties exhibited by bone tissue are of paramount importance as they could aid in explaining the effects of mechano-regulation and electrical stimulation on bone healing (Cerrolaza et al. 2017).

Several mathematical models of bone remodelling have been reported in the literature and most of them are based on the qualitative observations by Wolff (Wolff 1893). These models have been numerically implemented in finite element analysis to evaluate their predictive capacity to simulate bone adaptation to mechanical loading (Huiskes et al. 1987; Beaupré et al. 1990a, b; Weinans et al. 1992; Mullender et al. 1994; Adachi et al. 2001; Ruimerman et al. 2005; Fernández et al. 2010, 2012a; Garzón-Alvarado et al. 2012; Idhammad et al. 2013; Garijo et al. 2014; Mohaghegh et al. 2014; Mauck et al. 2016) and are reviewed elsewhere (Gerhard et al. 2009; Webster and Muller 2011; Amirouche and Bobko 2015). The theories of bone adaptation that have been developed in the past decades to predict changes in bone shape and density are based on strain (Turner et al. 1997; Wang et al. 2016), stresses (Gong et al. 2013), strain energy density (SED) (Huiskes et al. 1987; Weinans et al. 1992; Mullender et al. 1994; Fernández et al. 2010, 2012a; Garzón-Alvarado et al. 2012; Saeidi et al. 2020; Suárez et al. 2012), deformations (Papathanasopoulou et al. 2002), cell proliferation (Pivonka et al. 2008) and mechanical damage (Prendergast and Taylor 1994; Garcia-Aznar et al. 2005; Martínez et al. 2006; Hambli et al. 2011). Although there are many important aspects of bone remodelling, they are limited only to the study of bone response to a specific stimulus.

Previous studies have shown that human bone has frequency-dependent electric and dielectric properties (Bassett and Becker 1962; Bassett et al. 1964; Su et al. 2016) and these are significant not only as a hypothesised feedback mechanism for bone remodelling, but also in the context of therapeutic electrical stimulation for bone healing and repair (Bassett and Becker 1962; Bassett et al. 1977; RamtTherefore, the objective of our numerical study

ani 2008). In this context, Qu et al. (2006) have demonstrated that the electromagnetic field has an influence on the bone remodelling and healing process under the effect of mechanical and electrical stimuli. Beside these stimuli, the effect of thermal load on bone remodelling has been taken into consideration by Qin et al. (2005).

Piezoelectricity is the ability of a material, e.g. quartz crystals, to generate a voltage when subjected to mechanical stress and conversely, generate a mechanical response when subjected to an electric field or voltage (Abramovich 2016). Fukada and Yasuda (1957) first demonstrated that bone has piezoelectric properties, which has been confirmed in many other studies (Anderson and Eriksson 1970; Gjelsvik 1973; Guzelsu et al. 1978; Pienkowski and Pollack 1983; Isaacson and Bloebaum 2010).

Bone piezoelectricity supports the idea that the bone adaptation process can be explained by matrix piezoelectricity, a potential mechanism by which osteocytes, the mechanosensory cells, may detect areas of high stress (Mohammadkhah et al. 2019). This states that applied mechanical stress generates electrical charges in collagen fibres and then serves as a stimulus to osteoblasts (bone-forming cells) (Bassett and Becker 1962; Bassett et al. 1964). Based on the experimental findings, the streaming potential generated by fluid flow through the bone matrix was proposed as an alternative mechanism to matrix piezoelectricity and responsible for strain-generated potentials (Pienkowski and Pollack 1983). Different piezoelectric responses have been measured for dry bone attributed to matrix piezoelectricity (Yasuda 1954; Fukada and Yasuda 1957; Johnson et al. 1980) and for wet bone attributed to streaming potential (Pienkowski and Pollack 1983; Otter et al. 1988; Iannacone et al. 1979). However, the exact mechanisms for the piezoelectricity of bone tissue have not been understood completely so far (Wieland et al. 2015). Several mathematical and computational models of bone remodelling have been proposed but only a few of them have considered the piezoelectric properties of bone (Cerrolaza et al. 2017; Fernández et al. 2012a; Garzón-Alvarado et al. 2012; Qu et al. 2006; Beheshtiha et al. 2015; Duarte et al. 2018) and are recently reviewed in (Mohammadkhah et al. 2019).

The generation of piezoelectricity in bone is a complex process and in recent numerical studies, it has been shown to play a vital role in bone remodelling and adaptation (Cerrolaza et al. 2017; Fernández et al. 2012a). In our present study, the strain-adaptive bone remodelling model (Weinans et al. 1992) that couples the displacement and bone density is implemented in the finite element method (FEM) using the numerical algorithm proposed by Fernández et al. (2010). This model is based on the principle that remodelling is induced by the local mechanical signal, which triggers the regulating bone cells. These cells detect a mechanical stimulus and cause local bone adaptations based on its magnitude. It uses the bone density to characterise bone internal morphology. In addition to the above model, a piezoelectric strain-adaptive bone remodelling model proposed by Fernández et al. (2012a) is implemented into FEM, where electro-mechanical dependence for mechanical properties has been introduced in the strain-adaptive bone remodelling model. The validation of the simulation results was performed by qualitative and quantitative comparison between the predicted bone density and the values obtained from the computed tomography (CT) scan.

Therefore, the objective of our numerical study was to simulate the strain-adaptive bone remodelling of a human femur without and with considering the piezoelectric effect using an open-source software framework. The findings of this study can be used to understand the response of bone to electromechanical loadings and, in turn, design a protocol for therapeutic electrical stimulations to reduce bone loss in case of physical impairment or osteoporosis. The paper is outlined as follows: In Sect. 2, the finite element analysis of strain-adaptive bone remodelling models using the open-source software framework is presented, followed by the detailed description of applied boundary conditions. The simulation results are discussed and validated against the corresponding CT data and literature studies in Sect. 3. Also in this section, a parametric study for the effect of uniform initial bone density on the final density distribution is conducted. Finally, Sect. 4 provides a brief conclusion of the study.

## Material and methods

### Strain-adaptive bone remodelling

**Fig. 1:**
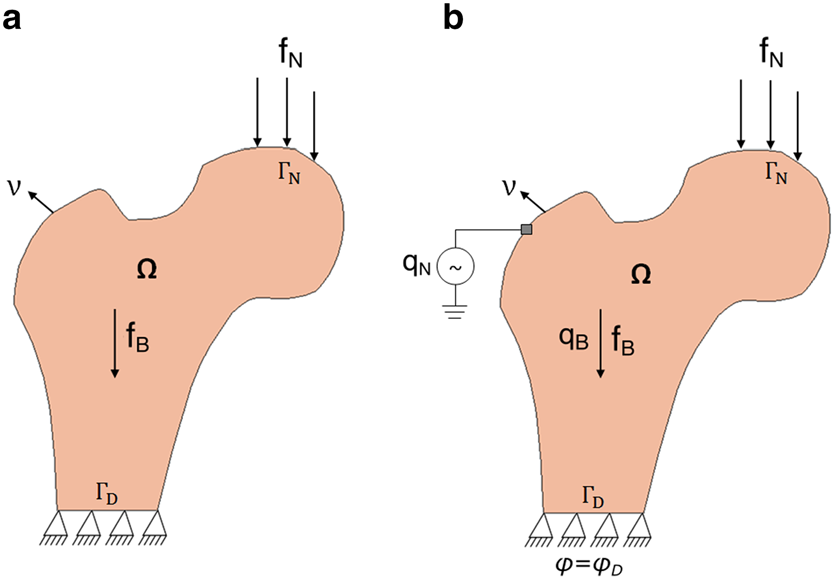
Strain-adaptive bone remodelling **a** without and **b** with the piezoelectric effect

Let $\varOmega$ be an open-bounded domain (see Fig. 1a) and its boundary is denoted by $\varGamma$=$\partial\varOmega$. This boundary was assumed to be Lipschitz continuous and it has been divided into two separate parts as Dirichlet boundary ${\varGamma }_{D}$ and Neumann boundary ${\varGamma }_{N}$. Here,${\varvec{f}}_{\varvec{B}}$ is the density of volume forces acting in the domain $\varOmega$ and ${\varvec{f}}_{\varvec{N}}$ is the density of traction forces that were applied on ${\varGamma }_{N}$. It has been assumed that the bone is clamped on ${\varGamma }_{D}$, i.e. here displacement vector $\varvec {u}$ = 0. Let [0,$T$] be the time interval of interest, where $T$ > 0 and $\nu \left(x\right)$ be the outward unit normal vector to $\varGamma$ at point $\varvec {x}$ (Fernández et al. 2010). Bold symbols represent vectors, tensors, or matrices. The linearised strain tensor $\varepsilon \left(u\right)$ is given by

1 $$ {{\varepsilon}}_{{{{ij}}}} \left( {{u}} \right) = \user2{ }\frac{1}{2}\user2{ }\left( {\frac{{\partial {{u}}_{{{i}}} }}{{\partial {{x}}_{{{j}}} }} + \user2{ }\frac{{\partial {{u}}_{{{j}}} }}{{\partial {{x}}_{{{i}}} }}} \right),\user2{ }i,j \, = \, 1, \ldots ,d, $$

where $\varvec {u}$ is the displacement field and *d* is the order of symmetric matrices (3 × 3). Here, the bone was considered to be linear-elastic and isotropic (Weinans et al. 1992) and the constitutive law for the stress field (N/mm^2^) can be given as follows:

2 $$ \varvec{\sigma } = \varvec{\sigma }\left( \varvec{u} \right) = 2\mu \left( \rho  \right)\varvec{\varepsilon }\left( \mathbf{u} \right) + ~\lambda \left( \rho  \right)~{\text{Div}}~\left( \varvec{u} \right)~\;{\varvec{I}}~\;{\text{in}}~~\bar{\Omega } \times \left[ {0,~T} \right], $$

where $\mu \left(\rho \right)$ and $\lambda \left(\rho \right)$ are Lamé coefficients of the material that were assumed to be dependent on the bone apparent density denoted by $\rho$, Div represents the divergence operator and I denotes the identity operator (Fernández et al. 2010). For the plane strain hypothesis or the three-dimensional case, Lamé coefficients can be expressed in terms of elastic modulus $E(\rho )$ and Poisson’s ratio $k\left(\rho \right)$ as follows:

3 $$\mu \left( \rho  \right) = ~\frac{{E\left( \rho  \right)}}{{2\left( {1 + k\left( \rho  \right)} \right)}}~\quad {\text{and}}\quad \lambda \left( \rho  \right) = ~\frac{{k\left( \rho  \right)E\left( \rho  \right)}}{{1 - k^{2} \left( \rho  \right)}}$$

The Poisson’s ratio was assumed to be independent of $\rho$ (and thus, $k\left(\rho \right)$ =$k$) and, the following equation was used for elastic modulus depending on the apparent density:

4 $$ E\left( \rho \right) = M\rho^{\gamma } , $$

where $M$ and $\gamma$ are positive constitutive constants that characterise bone behaviour (Weinans et al. 1992). The evolution of the apparent density function was obtained from the following first-order ordinary differential equation (Beaupré et al. 1990a; Weinans et al. 1992; Fernández et al. 2010, 2012a, b; Idhammad et al. 2013; Huiskes et al. 1989),

5 $$  \frac{{{{d}}\rho }}{{{{d}}t}} = B\left( {\frac{{U\left( {\sigma \left( u \right),~~\varepsilon \left( u \right)} \right)}}{\rho } - ~S_{r} } \right)~\;{\text{in}}\;\varOmega  \times \left( {0,~T} \right)  $$

where $B$ and ${S}_{r}$ are the experimental constants and their values are mentioned in Table 1. Further, the SED as mechanical stimulus $U(\sigma \left(u\right), \varepsilon (u))$ can be given as:

6 $$ U\left( {{\varvec{\sigma}}\left( {\varvec{u}} \right),{\varvec{\varepsilon}}\left( {\varvec{u}} \right)} \right) = \frac{1}{2} {\varvec{\sigma}}\left( {\varvec{u}} \right){ }:{\varvec{\varepsilon}}\left( {\varvec{u}} \right), $$

where ‘:’ denotes the inner product and it has been assumed that the apparent density function is bounded as:

7 $$ \rho_{a} \le \rho \le \rho_{b} , $$

where ${\rho }_{a}$ and ${\rho }_{b}$ represent the minimal and maximal density corresponding to the resorbed and the cortical bone, respectively. Neumann boundary conditions were applied to the femoral head and the greater trochanter acting in opposite directions as action and reaction forces, respectively, resulting from muscle activities. Applying Green’s formula, the weak form of the strain-adaptive bone remodelling problem can be given as:

8 $$ \int\limits_{\varOmega } {2\mu \left( \rho \right){\varvec{\varepsilon}}\left( {\varvec{u}} \right) : {\varvec{\varepsilon}}\left( {\varvec{v}} \right) + \lambda \left( \rho \right){\text{Tr}}\left( {{\varvec{\varepsilon}}\left( {\varvec{u}} \right)} \right){\text{Tr}}\left( {{\varvec{\varepsilon}}\left( {\varvec{v}} \right)} \right) d\user2{x } = \int\limits_{\varOmega } {{\varvec{f}}_{B} \left( t \right) \cdot {\varvec{v}} d{\varvec{x}} + } \int\limits_{{\varGamma_{N} }} {{\varvec{f}}_{N} \left( t \right) \cdot {\varvec{v}} d\varGamma } } , $$

where $\mathrm{Tr}$ denotes the classical trace operator, $v$ is the test function and $d{\varvec{x}}$ denotes the differential element for integration over the domain $\Omega$. The bone remodelling process was assumed to be quasi-static and thus, the effects of inertia were neglected. More details about this model can be found in (Fernández 2010; Fernández et al. 2010). For numerical analysis of strain-adaptive bone remodelling in the open-source finite element software FEniCS (www.fenicsproject.org, version 2019.1.0, GNU-GPL) (Logg et al. 2012; Alnæs et al. 2015), a vector function space with Lagrange elements of order 2 was used for the displacement field.

**Table 1:** Values for parameters used in the finite element simulations

Parameter	Name	Quantity	References
*ρ*_*a*_	Minimal bone density	0.010 g/cm^3^	(Weinans et al. 1992)
*ρ*_*b*_	Maximal bone density	1.740 g/cm^3^	(Weinans et al. 1992)
*S*_*r*_	Reference stimulus	0.004 J/g	(Weinans et al. 1992)
*B*	Experimental constant	1 (gcm^−3^)^2^ (MPa day)^−1^	(Weinans et al. 1992)
*M*	Constitutive constant	3790 MPa/(cm^3^/g)^2^	(Weinans et al. 1992)
$\gamma$	Constitutive constant	3	(Weinans et al. 1992)
${\varvec{f}}_{\varvec{B}}$	Body force	0 N/m^2^	(Fernández et al. 2012a)
*Piezoelectric coefficients*			
${e}_{31}$	Piezoelectric coefficient	1.50765 × 10^–9^ C/mm^2^	(Fernández et al. 2012a; Fotiadis et al. 1999)
${e}_{33}$	Piezoelectric coefficient	1.87209 × 10^–9^ C/mm^2^	(Fernández et al. 2012a; Fotiadis et al. 1999)
${e}_{15}$	Piezoelectric coefficient	3.57643 × 10^–9^ C/mm^2^	(Fernández et al. 2012a; Fotiadis et al. 1999)
*Electric permittivity coefficients*			
$\beta$_*11*_	Permittivity coefficient	88.54 × 10^–12^ F/mm	(Fernández et al. 2012a; Fotiadis et al. 1999)
$\beta$_33_	Permittivity coefficient	106.248 × 10^–12^ F/mm	(Fernández et al. 2012a; Fotiadis et al. 1999)

### Piezoelectric strain-adaptive bone remodelling

Several algorithms have been proposed to calculate the change in bone density under mechanical loadings; however, only a few of them have taken the piezoelectric effect of bone into account. Based on the proposed piezoelectric strain-adaptive bone remodelling algorithm by Fernández et al. (2012a), in addition to the parameters mentioned in Sect. 2.1, let ${q}_{B}$ be the density of volume electric charges present in domain $\varOmega$, ${q}_{N}$ be the density of surface electric charges applied on ${\varGamma }_{N}$ externally, $\varphi$ be the electric potential and an electric potential ${\varphi }_{D}=0$ was applied to the clamped boundary (see Fig. 1b) (Fernández et al. 2012a). Here, to impose the boundary conditions for the displacements and the electric potential, the same decomposition of the boundary has been used. For the piezoelectric bone remodelling, the constitutive law for the stress field (N/mm^2^) can be given as:

9 $$ {\sigma } = 2\mu \left( \rho  \right){\varepsilon }\left( {u} \right) + \lambda \left( \rho  \right){\text{Div}}\left( {u} \right){\text{I}}~ - ~\alpha \left( \rho  \right){\mathscr{E}}^{{*~}} {\mathbf{E}}\left( \varphi  \right)\;~{\text{in}}~\;\bar{\Omega }~ \times \left[ {0,T} \right],$$

where *α*(*ρ*) is a constitutive function, which was assumed to be dependent on the apparent density function similar to the elastic modulus and can be written as (Fernández et al. 2012a),

10 $$ {\upalpha }\left( \rho \right) = \rho^{\gamma } . $$

Further, $\mathscr {E}^*$ denotes the transpose of the third-order piezoelectric tensor $\mathscr {E}^*$described below and $\mathscr {E}^*$represents the stationary electric field, which, as a conservative field, can be calculated from the gradient of the electrostatic potential $\varphi$ (van Rienen 2001):

11 $$ {\mathbf{E}} = { } - \nabla \varphi $$

**Fig. 2:**
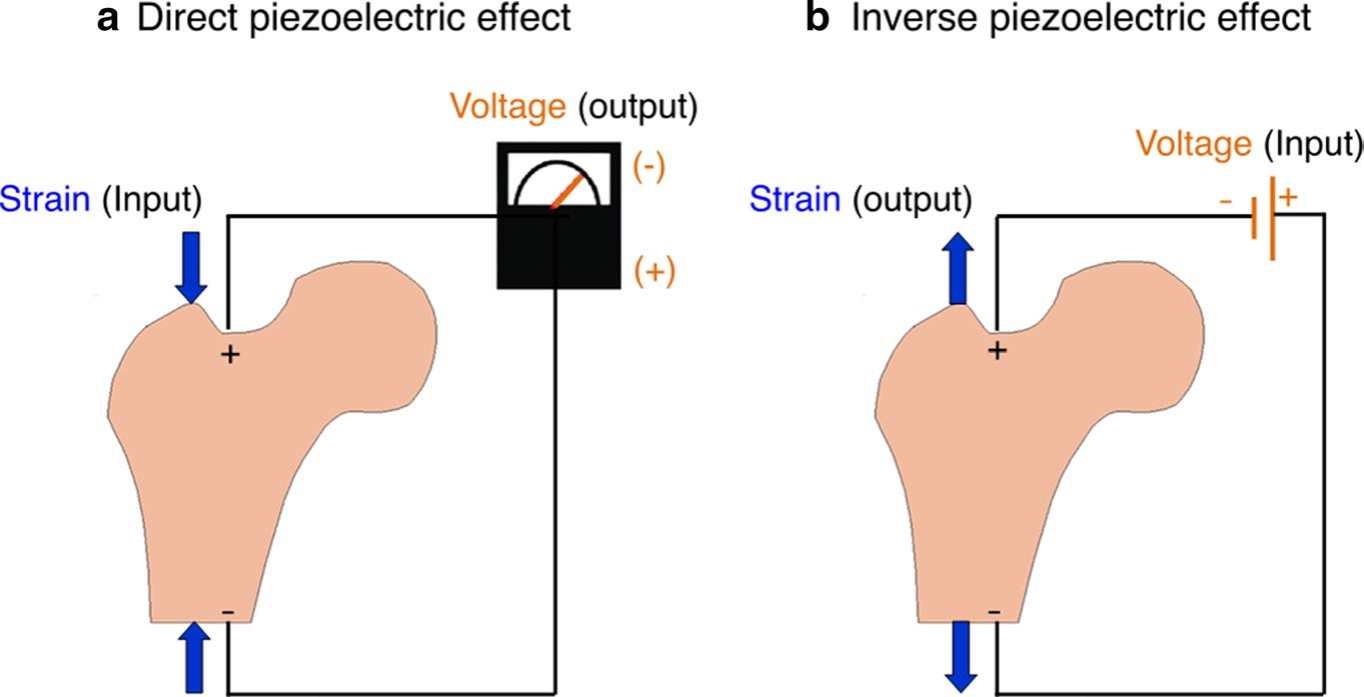
Schematic representation of **a** direct and **b** inverse piezoelectric effect

The constitutive law for the electric displacement field (C/mm^2^) can be given as:

12 $$ {\mathbf{D}} = {\upalpha }\left( \rho \right){\mathscr{E}} \varepsilon \left( u \right) + {\upalpha }\left( \rho \right)\varvec\beta {\mathbf{E}}\left( \varphi \right), $$

where $\varvec\beta$ is the electric permittivity tensor (Batra and Yang 1995). The constitutive equations for stress (Eq. (9)) and electric displacement field (Eq. (12)) define the piezoelectric effect of bone. When subjected to a mechanical load, it generates an electric charge (direct piezoelectric effect) and conversely, when an electrical charge is applied, strains/stresses can appear in bone (inverse piezoelectric effect) (Fernández et al. 2012a) (see Fig. 2).

**Fig. 3:**
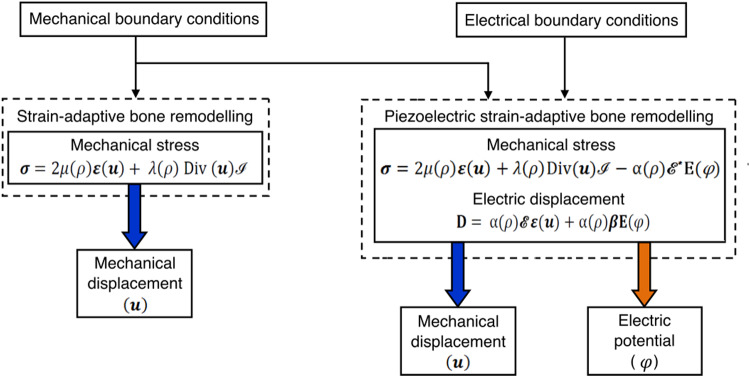
Simulation flow diagram

Similar to other authors (Fernández et al. 2012a, c; Qin and Ye 2004), the bone was assumed to behave like a crystal with hexagonal symmetry meaning that the third-order piezoelectric stress tensor $\mathscr{E}$ is defined by four values and the electric permittivity tensor (dielectric tensor)$\varvec\beta$ is a diagonal matrix with two constants. These tensors can be written in the following matrix form:

13 $$ {\mathscr{E }} = \left( {\begin{array}{*{20}c}    0 & 0 & 0  \\    0 & 0 & 0  \\    {e}_{{31}}  & {e}_{{31}}  & {e}_{{33}}   \\   \end{array} \begin{array}{*{20}c}    {e}_{{14}}  & {e}_{{15}}  & 0  \\    {e}_{{15}}  &  - {e}_{{14}}  & 0  \\    0 & 0 & 0  \\   \end{array} } \right)~\;{\text{and}}\;{\varvec\beta } = \left( {\begin{array}{*{20}c}    {\beta }_{{11}}  & 0 & 0  \\    0 & {\beta }_{{11}}  & 0  \\    0 & 0 & {\beta }_{{33}}   \\   \end{array} } \right), $$

where the third direction represents the longitudinal direction of the femur (Fernández et al. 2012c). In piezoelectric strain-adaptive bone remodelling, the displacement field $u$ and the electric potential $\varphi$ were obtained by solving the following coupled linear variational equations:

14 $$ \begin{gathered} \int\limits_{\varOmega } {2\mu \left( \rho \right)\varepsilon \left( u \right){ }: \varepsilon \left( v \right) + \lambda \left( \rho \right){\text{Tr}}\left( {\varepsilon \left( u \right)} \right){\text{Tr}}\left( {\varepsilon \left( v \right)} \right) dx} \hfill \\ = \int\limits_{\varOmega } {f_{B} \left( t \right) \cdot v dx + } \int\limits_{{\varGamma_{N} }} {f_{N} \left( t \right) \cdot v d\Gamma { } - \int\limits_{\varOmega } {(\rho \left( t \right)^{\varGamma } } } {\mathscr{E}}^{*} \nabla \varphi \left( t \right),{ }\varepsilon \left( v \right)) dx, \hfill \\ \end{gathered} $$

15 $$ \begin{gathered} \int\limits_{\varOmega } {(\rho \left( t \right)^{\gamma } \varvec\beta \nabla \varphi \left( t \right),} \nabla \psi ) dx \hfill \\ = \int\limits_{\varOmega } {q_{B} \left( t \right) \psi dx + } \int\limits_{{\varGamma_{N} }} {q_{N} \left( t \right) \psi d\varGamma } + \int\limits_{\varOmega } {(\rho \left( t \right)^{\gamma } }{\mathscr{E}}\varepsilon \left( {{\mathbf{u}}\left( t \right)} \right),\nabla \psi ) dx, \hfill \\ \end{gathered} $$

where $\psi$ is the test function, $dx$ denotes the differential element for integration over the domain $\varOmega$ and ${q}_{B}=\mathrm{div}{D}$. For simplicity, the formulation in this work was restricted to isothermal and quasi-static conditions and more details about this model can be found in (Fernández 2010; Fernández et al. 2012a). For numerical analysis of piezoelectric bone remodelling in the open-source finite element software FEniCS, a mixed-function space consisting of vector and scalar functions for the mechanical displacement and electric potential, respectively, was used with Lagrange elements of order 2. A block diagram of the numerical scheme implemented in this study is shown in Fig. 3.

Throughout this paper, the time derivatives were discretised using the forward (or explicit) Euler method as follows:

16 $$ \frac{\partial \rho }{{\partial t}} \approx \frac{{\rho_{n} - \rho_{n - 1} }}{\vartriangle t} , $$

where $\vartriangle t$ is the time-step size, ${\rho }_{n}$ and ${\rho }_{n-1}$ represent the bone density for the new and the current time step, respectively. The values of parameters used in numerical simulations are tabulated in Table 1.

### Open-source software framework to set up a finite element model

**Fig. 4:**
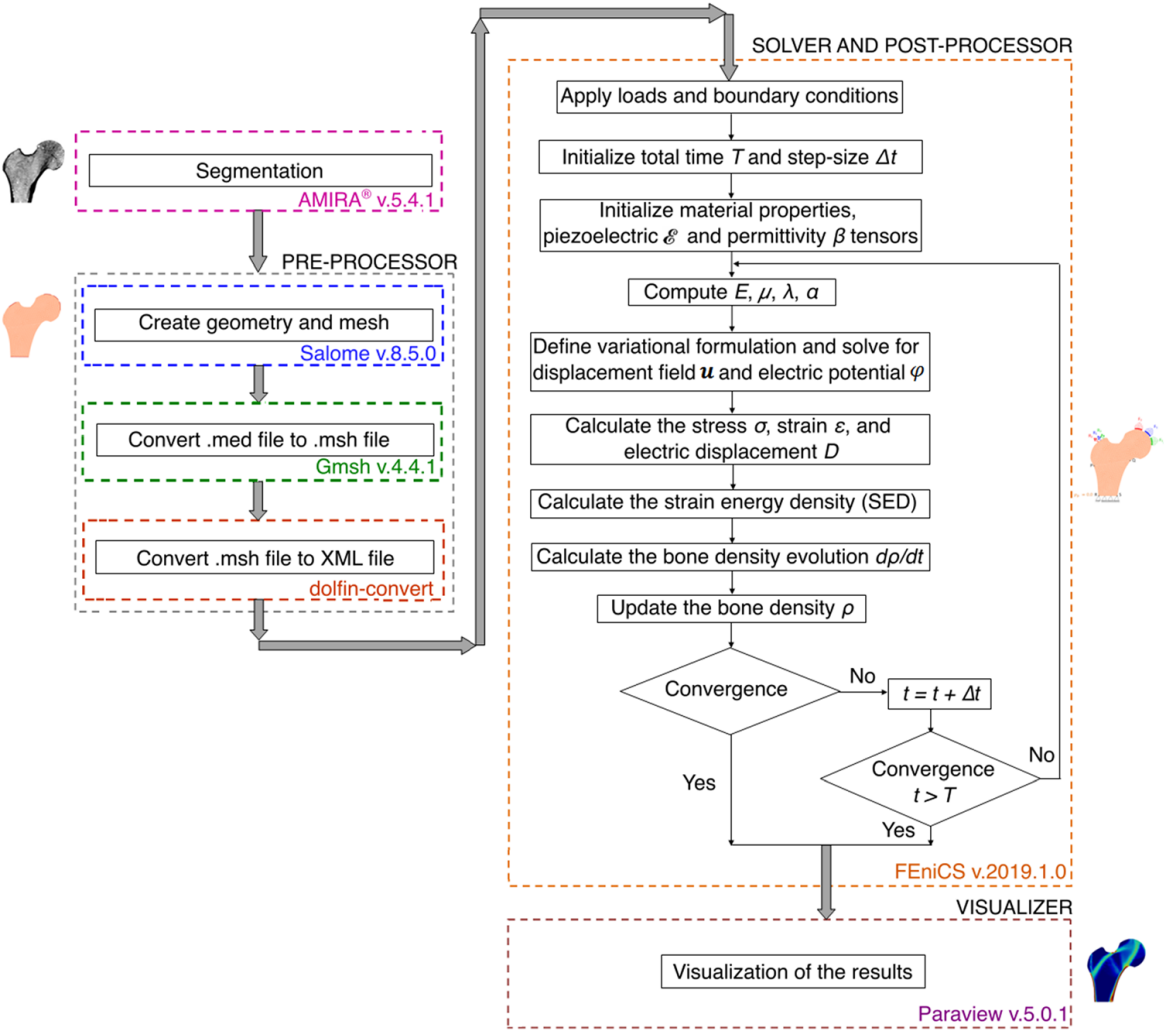
Schematic representation of the open- source software framework used for finite element simulations of piezoelectric strain-adaptive bone remodelling. Salome, Gmsh, Dolfin-convert, FEniCS and Paraview each are open-source software. In order to reuse our previous work, the commercial software AMIRA® was still used here, but it can be easily replaced by existing open-source software

Reproducibility of the results is an important general principle of scientific work. In the present study, with the aim to better understand the bone remodelling under electrical stimulation as a basis to design therapeutic strategies and electrically active implants, we used open-source software to ensure reproducibility. In our implementation of the open-source framework described in Fig. 4, we followed the principles of Open Science, i.e. to create transparency in numerical implementation, to make scientific data publicly available and to allow this data to be reused. In addition to enabling improved reproducibility, open-source software has several other advantages over commercial software. Open-source software provides high flexibility to customise the source code to meet specific study needs. Next, it is cost-effective, i.e. freely available with no maintenance fees. Further, open-source involves constant collaboration inputs from multiple developers and/or community members. Moreover, it is fairly easy to deploy across platforms. Nevertheless, there are also certain challenges in using open-source. Firstly, it lacks broad technical support and in depth documentation. Secondly, there can be unanticipated costs in terms of time and efforts associated with the modification of open-source software for user environment set-up. Finally, speed and scalability can also be a cause of concern when using open-source (Mogos 2020; DeVoe 2013).

Figure 4 represents a workflow of the main steps of piezoelectric strain-adaptive bone remodelling simulations using the open-source software framework adapted from Abali (2016). To carry out these simulations, the open-source software packages used were Salome (www.salome-platform.org, version 8.5.0, GNU-LGPL) (Ribes et al. 2007), Gmsh (http://gmsh.info/, version 4.4.1, GNU-GPL) (Geuzaine and Remacle 2009) and dolfin-convert (GNU-LGPL) as pre-processors, the Python-based open-source software FEniCS (Logg et al. 2012; Alnæs et al. 2015) as solver and post-processor and Paraview (https://www.paraview.org/, version 5.0.1, 3-Clause BSD License) (Ahrens et al. 2005) as a visualisation tool.

The steps executed to set up the finite element model were as follows: The medical image processing software AMIRA® (FEI Visualization Sciences Group, Hillsboro, OR, USA, version 5.4.1) was used for segmentation of femur and the resulting set of points describes the contour of the bone. Further, using these points, the femur geometry and sub-domains were created and meshed in open-source Salome platform. The numerical analysis of bone remodelling problem was performed using FEniCS, which contains programming and mathematical tools for solving partial differential equations (PDEs) with FEM. Although the geometry and meshing capabilities of this software are very limited, it can import this information from other open-source meshing software. The open-source meshing software Gmsh was thus used to convert the Salome mesh file with.med extension to.msh file. Subsequently, the Python script dolfin-convert was used to convert this.msh file into the XML file, which is the preferred file format for FEniCS. After applying the appropriate loading and boundary conditions, the variational problem was solved and the necessary post-processing was performed in FEniCS (Bansod and van Rienen 2019). The box highlighted in orange (see Fig. 4) demonstrates the schematic representation of steps involved in piezoelectric strain-adaptive bone remodelling algorithm implemented in this study. Finally, the simulation results were visualised using Paraview software. In order to reuse our previous work, the commercial software AMIRA® was used here for bone segmentation but the use of this software is not mandatory. To comply with open-source philosophy, several open-source image segmentation tools such as ITK-SNAP (Sheppard et al. 2019), Seg3D (Venäläinen et al. 2016), ImageJ (Kim et al. 2016), or MITK (Nickel et al. 2018) are available to perform this task. The Python scripts and other files created for this study are available for open access on the GitHub repository (https://github.com/YDBansod/Bone_Remodelling).

### Mechanical boundary conditions

**Fig. 5:**
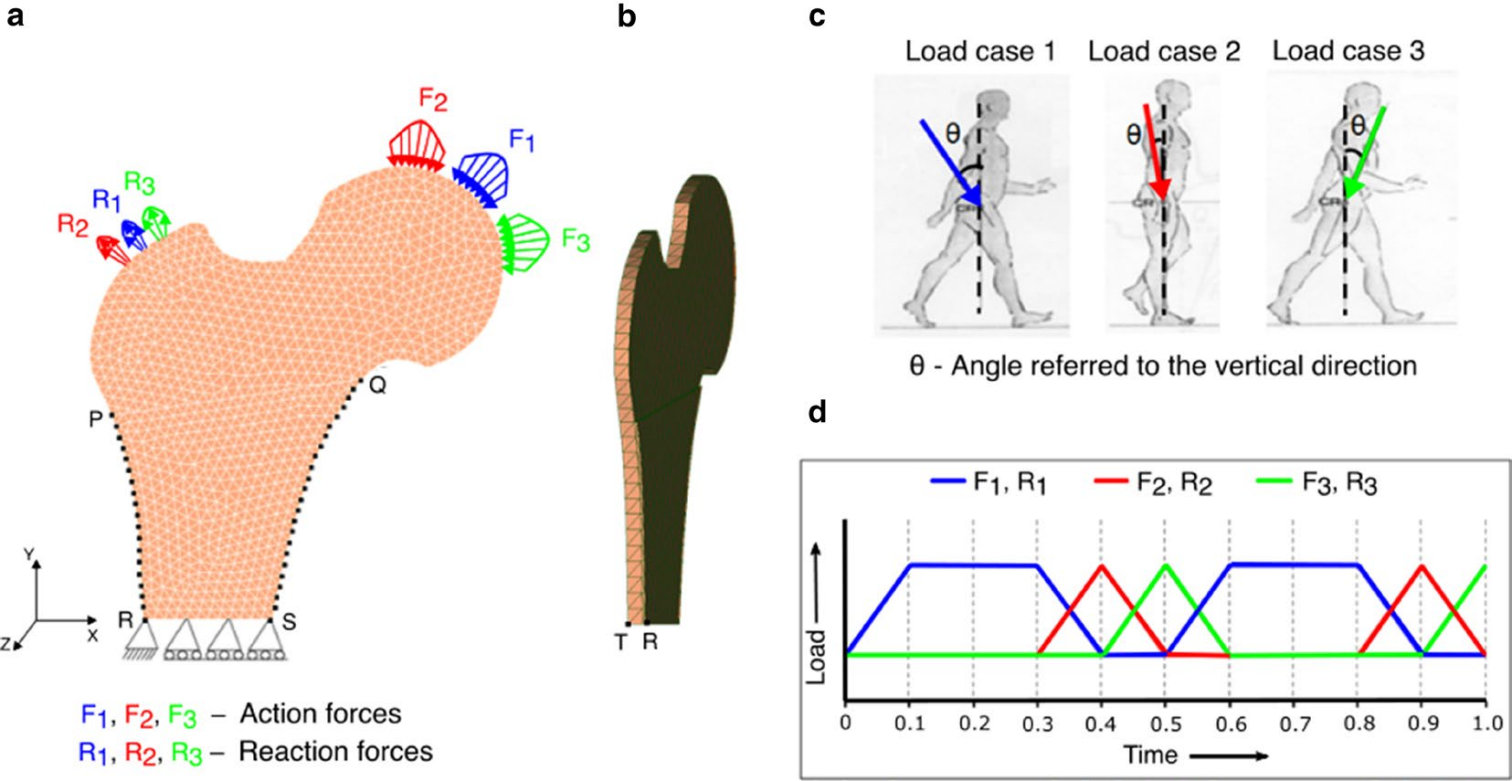
**a** Meshed finite element model of the human proximal femur and side-plate (joined at nodes highlighted by black dots) with applied mechanical boundary conditions (front-view); **b** lateral view of the proximal femur; **c** sequence of the gait cycle (Beaupré et al. 1990b; Jacobs et al. 1995); and **d** corresponding load pattern applied simultaneously and sequentially to the finite element model

A classical benchmark problem of the human proximal femur was used to simulate the strain-adaptive bone remodelling without and with the piezoelectric effect using an open-source framework. Figure 5a illustrates the finite element model of the proximal femur after meshing with 10-node isoparametric tetrahedral elements. More precisely, it consists of 5,233 elements and 1,875 nodes. Regarding the geometrical dimensions of bone, the distance between points P and Q is 66.59 mm and between points R and S (representing the diameter of the diaphysis) is 34.10 mm. In order to overcome the lack of connection between the two cortical layers of the diaphysis, similar to other authors (Beaupré et al. 1990a, b; Weinans et al. 1992; Fernández et al. 2010; Garijo et al. 2014; Nackenhorst 1997; Carter et al. 1989), an additional side-plate was considered joining these layers only at lateral nodes (along the edge P-R) and medial nodes (along the edge Q-S) marked with black dots (see Fig. 5a). The side-plate is represented by the four points P, R, S and Q. Furthermore, it consists of 2,009 elements and 743 nodes. The mechanical properties of the plate were considered to be similar to the cortical bone with an elastic modulus of 17,000 MPa (Weinans et al. 1992) and a Poisson’s ratio of 0.3 (Wirtz et al. 2000). In addition, its remodelling capacities were constrained and properties were assumed to be constant in time and space. In the present study, to execute matrix multiplication of tensors in FEniCS, the femur and the side-plate had to be modelled as slice with a uniform thickness of 1 mm and 0.1 mm, respectively. The domains were meshed using 10-node isoparametric tetrahedral elements with only one element in the transverse direction (see Fig. 5b) as FEniCS does not support hexahedral mesh. Dirichlet boundary conditions were applied at the lower part of the femur (see Fig. 5a), where the bottom face was restrained in the vertical direction and the left-most edge T-R (see Fig. 5b) was constrained in both vertical and horizontal directions to prevent rigid body motion during the analysis. To impose plane strain conditions (Fernández et al. 2010, 2012a), the front and back faces of both the femur and the side-plate were constrained in the z-direction (corresponding to the anterior–posterior direction). The total simulation time was set to be *T* = 300 days (remodelling period) with a time-step size of $\vartriangle t$ = 0.1 day.

Implementing the same scheme as described in (Beaupré et al. 1990b; Jacobs et al. 1995; Jacobs 1994), the remodelling behaviour was considered under the action of three simultaneous load cases that characterise the total load-time history for walking activity. Each load case consists of a set of parabolic distributed loads acting on the femoral head and the corresponding reaction forces induced by the abductor muscles acting on the greater trochanter (see Table 2). The first load case represents the moment when the foot touches the ground, while the other two represent the alternative moments of an exemplary gait cycle (see Fig. 5c) (Beaupré et al. 1990a). These cyclic loading cases with different frequencies were applied simultaneously in a sequential manner (see Fig. 5d), where each iteration represents one day.

**Table 2:** Mean value and angular orientation of the resultant forces for the three load cases considered in (Beaupré et al. 1990b; Jacobs et al. 1995). Angles are referred to the vertical direction (see Fig. 5c)

Load case	Load (*F*) applied on the femoral head		Reaction force (*R*) applied on the greater trochanter	
	Load (N)	Angle θ(°)	Load (N)	Angle θ(°)
1	2317	24	703	28
2	1158	− 15	351	− 8
3	1548	56	468	35

### Electrical boundary conditions

**Fig. 6:**
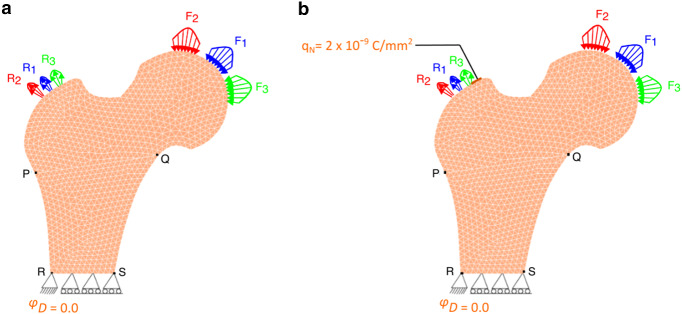
**a** Meshed finite element model of the proximal femur with applied mechanical and electrical boundary conditions simulating piezoelectric strain-adaptive bone remodelling; **b** the therapeutic electrical stimulation (surface charge) applied to the greater trochanter

To simulate the piezoelectric strain-adaptive bone remodelling, the loading and boundary conditions are depicted in Fig. 6a. The previously used mechanical boundary conditions (Sect. 2.4) were supplemented with an additional electrical constraint applied at the left-most edge of the femur, where it was electrically grounded.

For a systematic comparison of the density distribution obtained from the strain-adaptive bone remodelling (Sect. 2.1), the piezoelectric bone remodelling simulation (Sect. 2.2) was initially run for *T* = 300 days. This also enables a fair comparison with similar literature studies (Fernández et al. 2012a). With the obtained density configuration as an initial state, it was further assumed that the daily physical activity of the person is reduced between days 300–400 and this has been incorporated in our simulations by applying the mechanical loads only once in three days for this duration referred to as reduced physical activity (Fernández et al. 2012a). When an electric stimulation is applied to piezoelectric material an associated mechanical displacement is obtained and in the case of the femur, a change in bone density is observed. Accordingly, similar to other studies (Cerrolaza et al. 2017; Fernández et al. 2012a), during the period of reduced physical activity, i.e. 300–400 days, a surface electric charge ${q}_{N}$ of 2 × 10^–9^ C/mm^2^ was applied to the greater trochanter (see Fig. 6b).

For both models of bone remodelling, i.e. without and with piezoelectric effect, simulations start with a uniform initial density ${\rho }_{0}$ of 0.8 g/cm^3^ (Weinans et al. 1992; Fernández et al. 2010, 2012a) and by applying the appropriate boundary conditions, changes in the bone density distribution were computed. The initial bone density of 0.8 g/cm^3^ was chosen as it represents a near average of the minimal and maximal bone densities considered in the remodelling algorithms (Marzban et al. 2012). Moreover, this also allows a fair comparison of the obtained simulation results with those from the literature (Fernández et al. 2010, 2012a; Jacobs et al. 1995; Weinans et al. 1992). To study the effect of the chosen initial bone density on the final density distribution, a range of initial density values from 0.2 to 1.4 g/cm^3^, with an increment of 0.3 g/cm^3^ was considered. Additionally, the variation of ± 5% of the initial bone density of 0.8 g/cm^3^ that leads to the values of 0.76 g/cm^3^ and 0.84 g/cm^3^, respectively, was considered to evaluate the model performance. While studying the effect of initial bone density, the other simulation parameters remained unchanged. During the simulations, bone adapts its structure in response to the externally applied mechanical stimuli and exhibits a heterogeneous density distribution at the final time. The finite element simulations were performed under the assumption of small displacement theory and homogeneous, isotropic linear-elastic material behaviour. To calculate the evolution of apparent bone density, an element-based approach was implemented, where the density was assumed to be elementwise constant. In the present study, time was discretised using forward Euler method with a constant time-step size of 0.1 and a direct solver based on sparse LU decomposition (Gaussian elimination) was used to solve linear asymmetric system. Although not shown here, mesh and time convergence studies were performed and it was found that the results were independent of element and time-step size below 1 mm and 0.1 time units, respectively. The finite element simulations were performed on a workstation with 256 GB RAM, Intel(R) Xeon(R) CPU E5-2687Wv4@3.00 GHz with 4 cores using FEniCS and the strain-adaptive bone remodelling took approximately 10 s and the same with piezoelectric effect required about 14 s for each time-step iteration.

### Conversion of computed tomography (CT) HU values into BMD using a calibration phantom

A human femur specimen of a 58 years male donor with no history of major injury or orthopaedic surgery was CT scanned (SOMATOM Definition AS + CT scanner, Siemens AG, Erlangen, Germany) with a calibration phantom (European Forearm Phantom-05–83, QRM GmbH, Möhrendorf, Germany) and the images were stored in DICOM format (Kluess et al. 2019). AMIRA® software was deployed to import these images and reconstruct the bone surface. Several experimental relationships are available in the literature relating the HU values with the bone density and other mechanical characteristics (Wirtz et al. 2000; Rho et al. 1995; Keller 1994; Lotz et al. 1990; Peng et al. 2007). In the present study, a calibration phantom was used to associate the HU values with the ash density of the femoral bone (${\rho }_{ash}= HU/895.93$) and more details can be found in our previous study (Geier et al. 2019). Subsequently, to compare the density values from the CT scan with the values from the FE model, the bone ash density was converted into the apparent density ${\rho }_{app}$ (dry bone) using the relation ${\rho }_{ash}/{\rho }_{app}= 0.55$ (Keyak et al. 1994; Schileo et al. 2008). The procedure used in this study was approved by the ethical review committee (Ethics Committee of the Bavarian Medical Association, BLAEK 2011–058, Munich, Germany).

To validate the simulation results, the predicted bone density distributions were compared qualitatively and quantitatively with the CT data. For the qualitative analysis, the predicted bone density distribution was visually compared with the CT scan of the same femur. In the case of the quantitative analysis, root-mean-square (RMS) error, based on absolute differences between the bone apparent density of the simulation and of the CT scan image, were computed, along with the mean deviation of the differences. Using the nodal coordinates obtained from the finite element model, the same locations on the referent CT were chosen to calculate the corresponding BMD values.

## Results and discussion

### Strain-adaptive bone remodelling without and with the piezoelectric effect

**Fig. 7:**
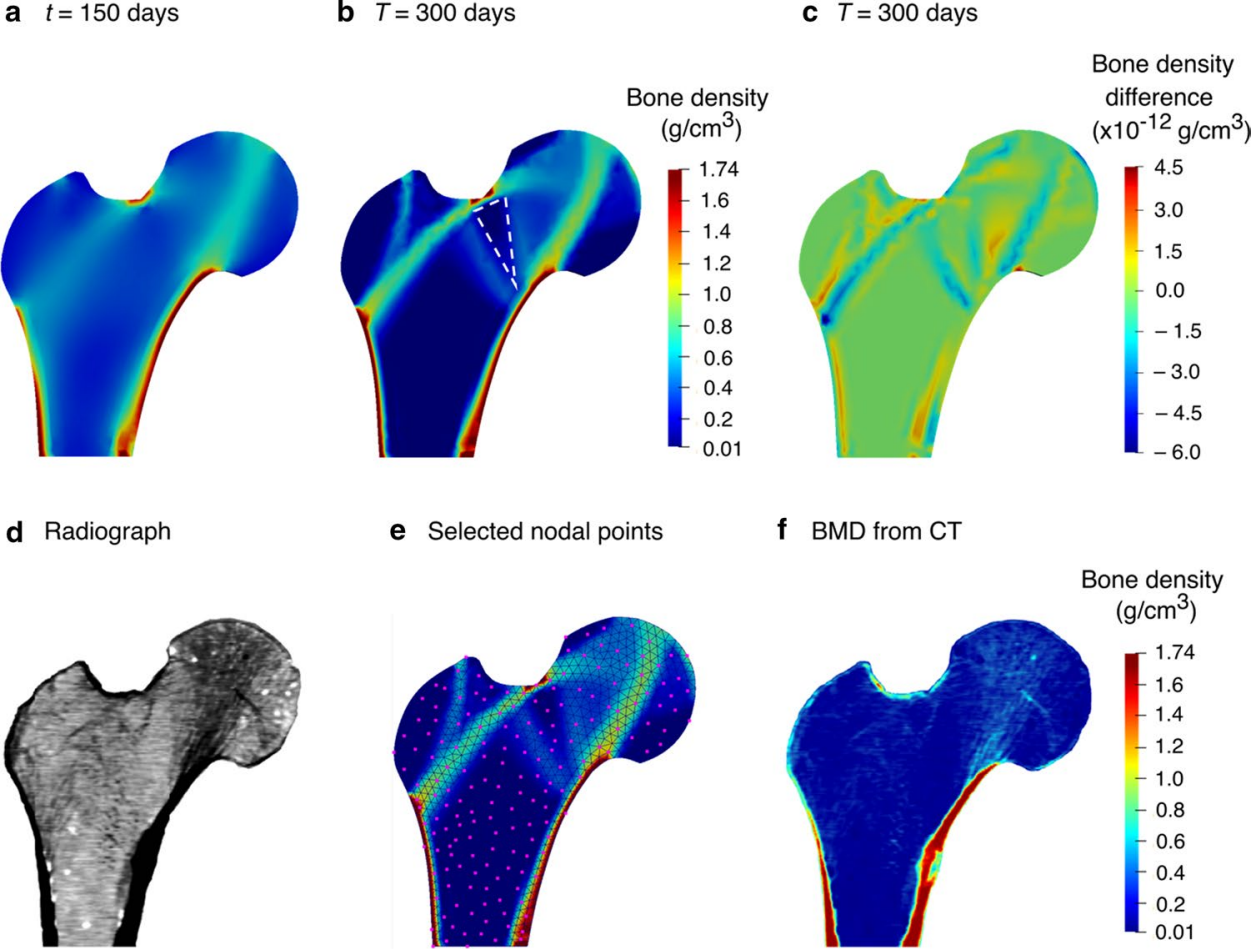
For strain-adaptive bone remodelling, the evolution of bone density at **a** intermediate time *t* = 150 days and **b** final time *T* = 300 days; **c** differences between bone density without and with considering the piezoelectric effect at *T* = 300 days; **d** qualitative comparison with the radiograph of the proximal femur; **e** position of manually selected 176 random nodal points for calculating the RMS and mean deviation; **f** quantitative comparison with the CT bone density

Starting from a homogeneous density distribution, the bone densities predicted by the strain-adaptive bone remodelling model (Sect. 2.1) at intermediate time *t* = 150 days (Fig. 7a) and final time *T* = 300 days (Fig. 7b) using an interpolation post-processing technique are shown in Fig. 7a-b. To explore the influence of additional electrical stimulation on the mechanically loaded human femur, the piezoelectric strain-adaptive bone remodelling model (Sect. 2.2) was also simulated. For quantitative comparison of both these models (i.e. the strain-adaptive bone remodelling model (Sect. 2.1) and the piezoelectric strain-adaptive bone remodelling model (Sect. 2.2)), the difference between the predicted final bone densities is plotted in Fig. 7c. It was observed that the simulation results are very similar, with minor variations in the trabecular trajectories. Thus, it is evident that the piezoelectric potential does not have much influence on the distribution of bone density and this could be because the electrical potential generated due to walking activity is very low (Fernández et al. 2012a).

For qualitative analysis of simulation results, the predicted bone density distribution (Fig. 7b) was visually compared to the radiographic scan of the proximal human femur (see Fig. 7d). The final configuration predicted a reasonably accurate density distribution with an intramedullary canal and cortical wall, Ward’s triangle (highlighted with white dashed lines) and an internal trabecular pattern of the femoral head. Also, these results are in good agreement with other bone remodelling studies without and with considering piezoelectric effect performed using commercial software (Weinans et al. 1992; Nackenhorst 1997, 2018; Fernández et al. 2010, 2012a; Garijo et al. 2014; Jacobs et al. 1995; Villette and Phillips 2017; Cerrolaza et al. 2017; Pettermann et al. 1997) and this serves as a preliminary validation of the bone remodelling simulations using the open-source framework shown in Fig. 4. It is evident in several clinical studies (Zhang et al. 2020; Hauger et al. 2020; Gupta et al. 2020; Petrovic et al. 2019; Nilsson et al. 2019; Wishart et al. 1995) that bone loss appears to accelerate with increasing age. In this context, compared to the radiograph used in this study (Fig. 7d), the primary tensile trabeculae (convex curve directed towards the greater trochanter) is more prominent in the final bone density distribution (Fig. 7b) and the reason for this could be that the radiograph belongs to a relatively old man of 58 years.

For quantitative analysis of the simulation results, RMS error and mean deviation were computed for manually selected 176 random nodal points (highlighted with pink dots in Fig. 7e). A non-probability convenience sampling technique (Lombardo et al. 2017) was used to select these nodal points to cover the whole domain area rather than capturing all domain characteristics, which may yield skewed results. Although not shown here, the range of HU values (-200 to 1500 HU) obtained from the CT data corresponds well with those obtained by Perez et al. (2007, 2010), where high values were noticed in the femoral cortex. Figure 7f shows the bone density calculated using the HU-density relationship mentioned in Sect. 2.6. Comparing the predicted (Fig. 7b) and CT bone densities (Fig. 7f), the RMS error and mean deviation were calculated to be 0.218 g/cm^3^ and 0.220 g/cm^3^, respectively. These quantitative measurements can serve as additional validation of the implemented bone remodelling algorithms using an open-source framework. For better correlation between simulation results and CT observations, patient-specific initial bone density distribution, three-dimensional (3D) geometry, boundary conditions, or a combination of these could be taken into account. The simulation results presented here can be considered to be representative of the femur in general.

### Effect of the initial bone density

**Fig. 8:**
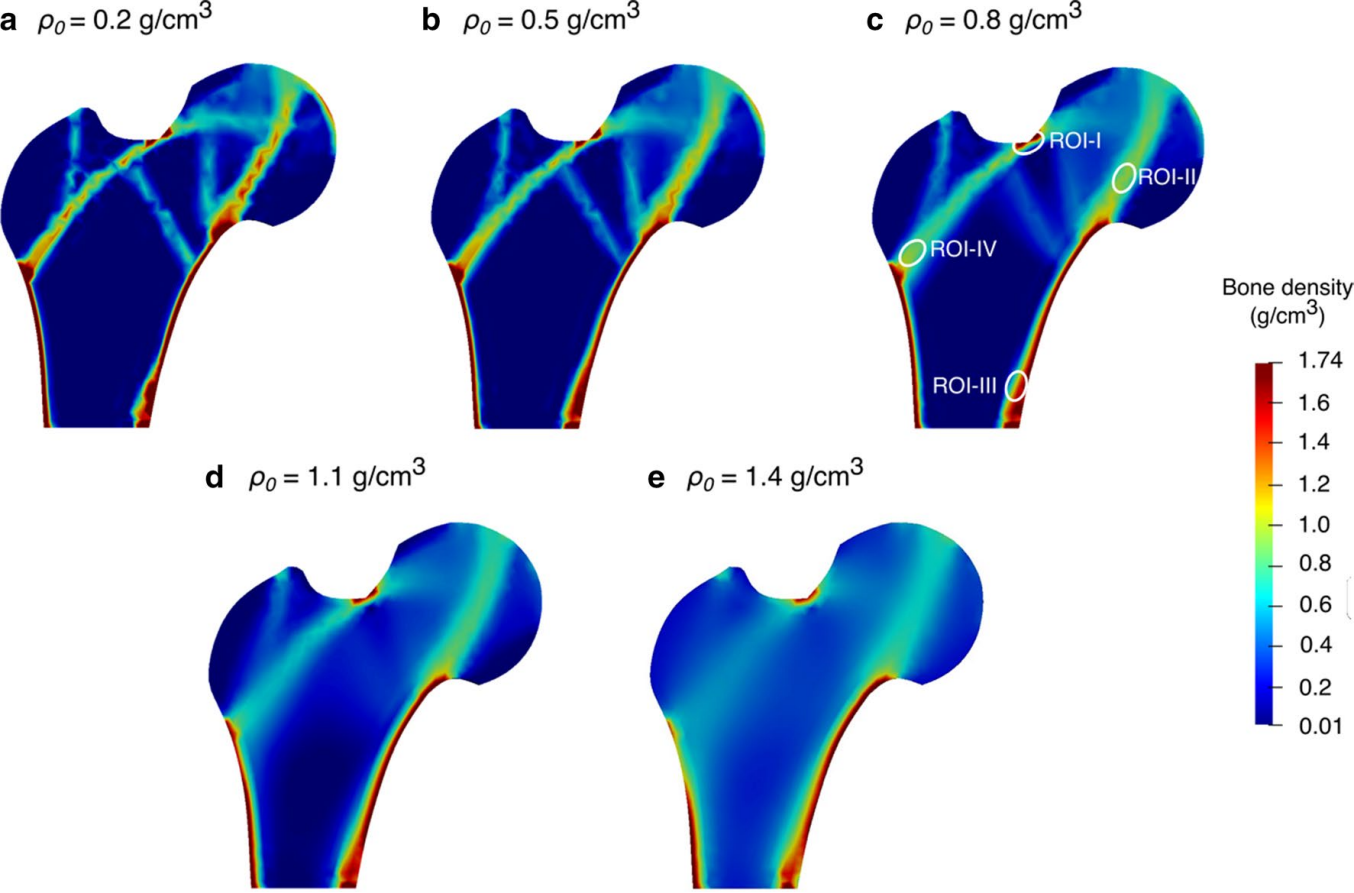
Bone density distributions predicted at the final time *T* = 300 days for different uniform initial densities **a** ${\rho }_{0}$ = 0.2 g/cm^3^, **b** ${\rho }_{0}$ = 0.5 g/cm^3^, **c** ${\rho }_{0}$ = 0.8 g/cm^3^ (with white ellipses highlighting regions of interest (ROIs)), **d** ${\rho }_{0}$ = 1.1 g/cm^3^ and **e** ${\rho }_{0}$ = 1.4 g/cm^3^

Several models using different initial uniform density values resulted in similar but not exactly the same bone density distribution at the final time. To study the influence of varying initial bone density on the final density distribution, the strain-adaptive bone remodelling was analysed for five different initial densities (${\rho }_{0}$) of 0.2, 0.5, 0.8, 1.1 and 1.4 g/cm^3^. The simulation results obtained are depicted in Fig. 8. It was observed that the final bone density distributions are not completely identical and depend on the initial density value. For the element-based remodelling algorithm implemented here, similar findings have been reported in literature studies (Turner et al. 1993; Weinans et al. 1989). For the node-based bone remodelling simulations, the effect of different initial bone densities was analysed by Fischer et al. (1997).

**Fig. 9:**
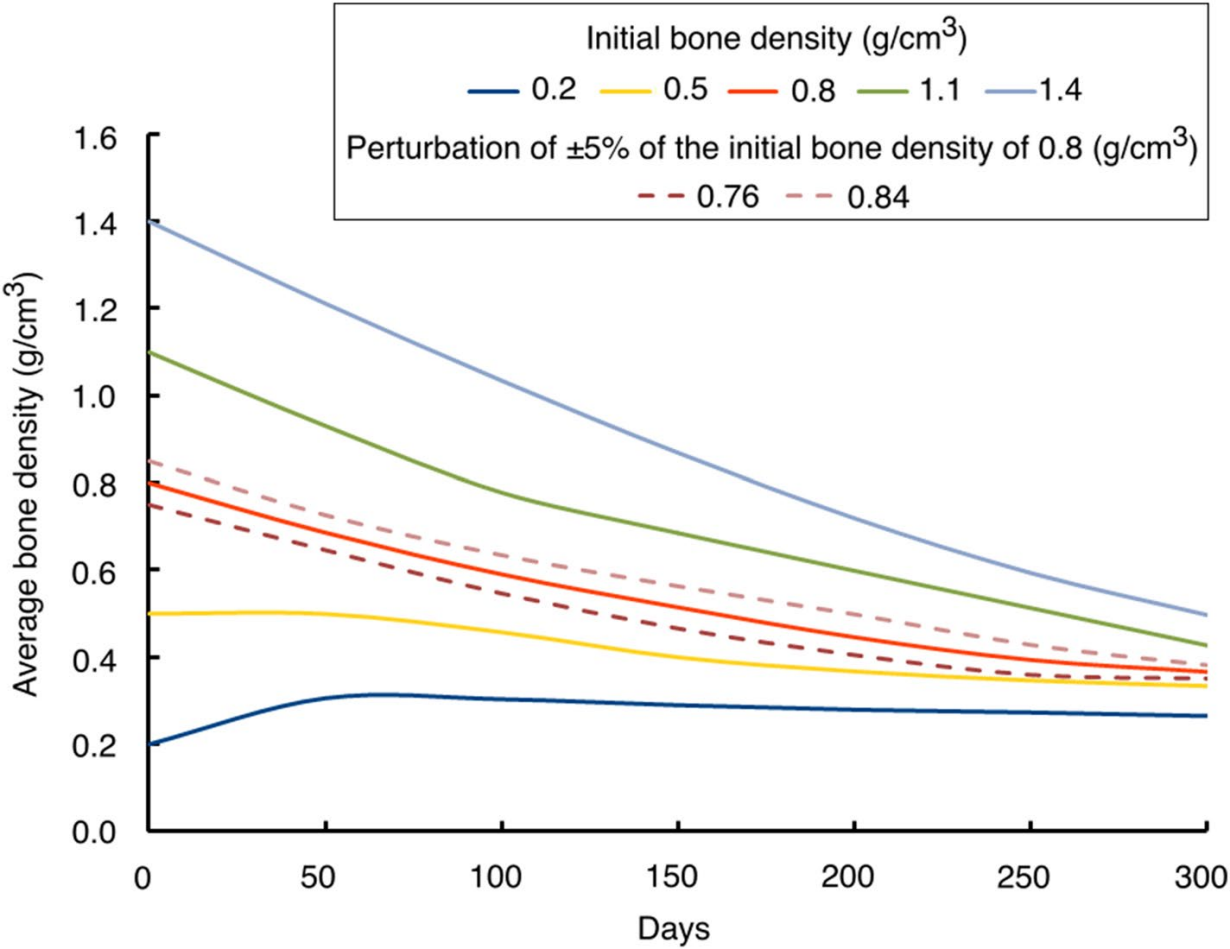
Evolution of average bone density for uniform initial density values (solid lines) ranging from 0.2 to 1.4 g/cm^3^ and with small perturbation (dashed lines) added to 0.8 g/cm^3^

When the initial bone density was greater than or equal to 0.8 g/cm^3^, the average bone density generally decreased over the remodelling period (see Fig. 9). On the other hand, when the initial bone density was lower than or equal to 0.5 g/cm^3^, the average bone density increased in the first 50 days and then decreased gradually. As time increased, the differences between average bone densities resulting from different initial density values were getting smaller. The average bone density at 0th day ranged from 0.2 to 1.4 g/cm^3^, at the 150th day was 0.28–0.86 g/cm^3^ and at the 300th day was 0.26–0.51 g/cm^3^. At the 300th day, the relative difference between the average bone densities is approximately within the range of 4.0%–23.0% and the distribution of bone densities resulting from different initial density values are also dissimilar (see Fig. 8). When a perturbation of ± 5% was added to the chosen uniform initial density of 0.8 g/cm^3^, the final bone density distribution obtained was similar but not the same (see dashed lines in Fig. 9). Therefore, it was evident that both bone density distribution (Fig. 8) and average bone density (Fig. 9) are dependent on the initial density values. However, the final density distribution should not be dependent on the initial conditions as they have not been obtained from experimental data and are mere numerical assumptions. In order to mitigate such dependency, the equilibrium zone (lazy zone) along with the saturated density change rate (Su et al. 2019; Martínez-Reina et al. 2016) could be included in the implemented bone remodelling algorithms. The lazy zone is a range of SED where the bone density does not change under a given loading condition and the saturated density change rate limits the bone response under disuse and overload. With increasing initial bone density, the area with the highest bone density (red) was reduced, but the area with the lowest bone density (blue) was enlarged (see Fig. 8), which resulted in lower overall average density and was consistent with the trends observed in Fig. 9.

**Fig. 10:**
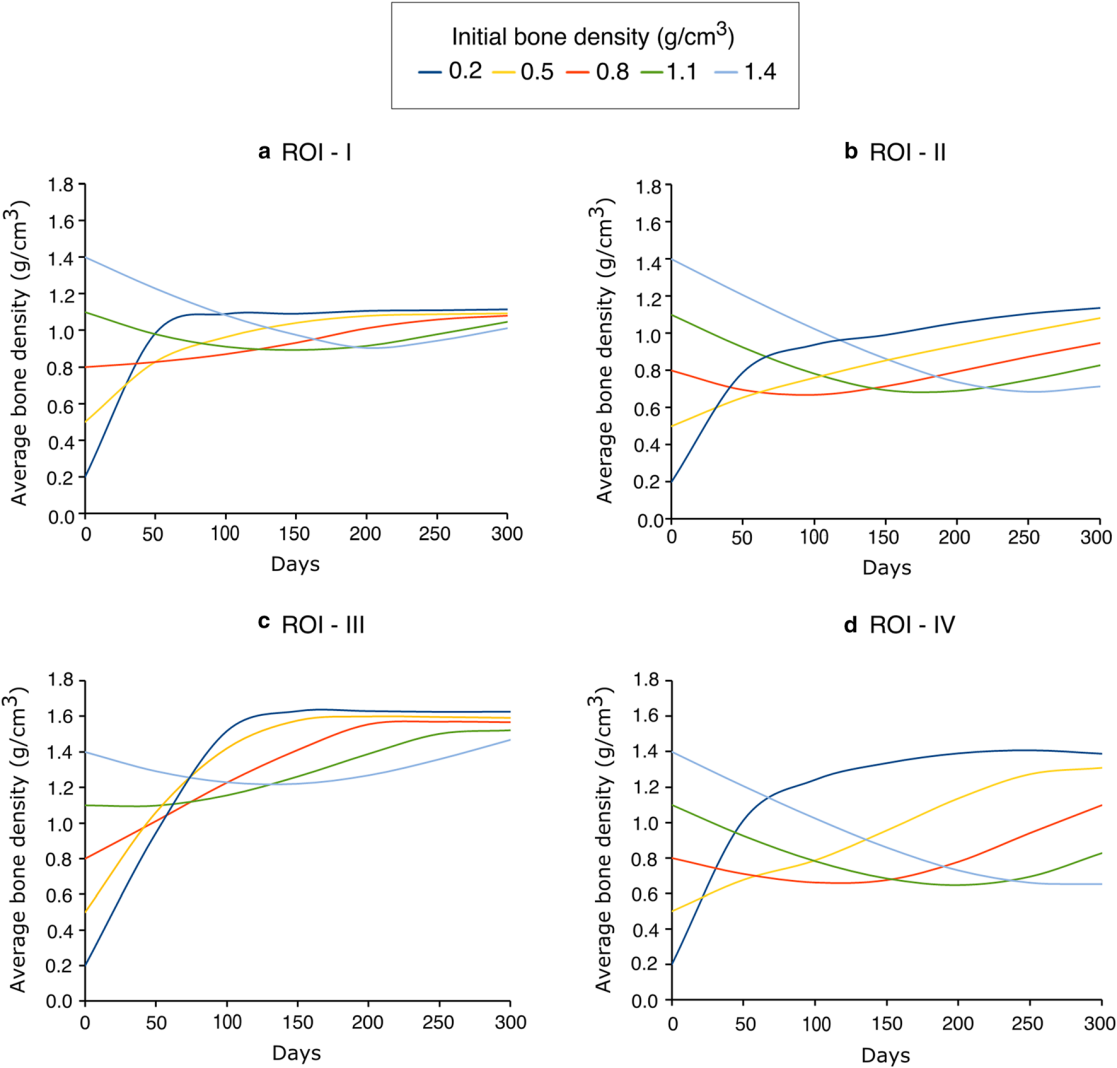
Evolution of average bone density in local regions of interest (ROIs) (highlighted in Fig. 8c) over time: (**a**) I (**b**) II (**c**) III and (**d**) IV

Further to study the local bone adaptation, the evolution of average bone density in local regions of interest (marked with white ellipses in Fig. 8c) is plotted in Fig. 10. For all ROIs, when the initial bone density was greater than 0.8 g/cm^3^, the average bone density decreased over time. On the other hand, when the initial bone density was lower than 0.8 g/cm^3^, the average bone density increased rapidly for the first few days and then changed slowly after 100 days.

Moreover, a lower initial density value of 0.2 g/cm^3^ resulted in the highest average bone density. For high strain regions (i.e. ROIs I and III), as days increased, the differences in average bone density resulting from different initial bone densities were getting smaller (see Fig. 10a and Fig. 10c). At the final time, i.e. 300th day, the differences in the average bone density were negligible for these regions and the distribution of bone density resulting from different initial bone densities were also similar (see Fig. 8). Additionally, a tendency of convergence to a common value is remarkable. For medium strain regions (i.e. ROIs II and IV), the average bone density resulting from different initial bone densities diverged as the time passed (see Fig. 10b and Fig. 10d).

The results of this study showed the adaptation process of the proximal femur bone under physiological loadings during the remodelling period. However, these simulation results are based on the assumption of uniform initial density, which is not the case in reality. Therefore, while interpreting the results, it is more important to focus on the final density distribution rather than the bone density adaptation process during the remodelling period. Starting from unrealistic uniform initial density assumption could be more appropriate for the study of numerical algorithms (Su et al. 2019; Nutu 2018). A sensitivity analysis was also conducted to investigate the influence of the reference stimulus on the predicted bone density distribution and the piezoelectric and permittivity tensors on the generated electric potentials. This analysis demonstrated that the piezoelectric strain-adaptive bone remodelling was sensitive to the values of these parameters.

### Piezoelectricity of bone tissue

**Fig. 11:**
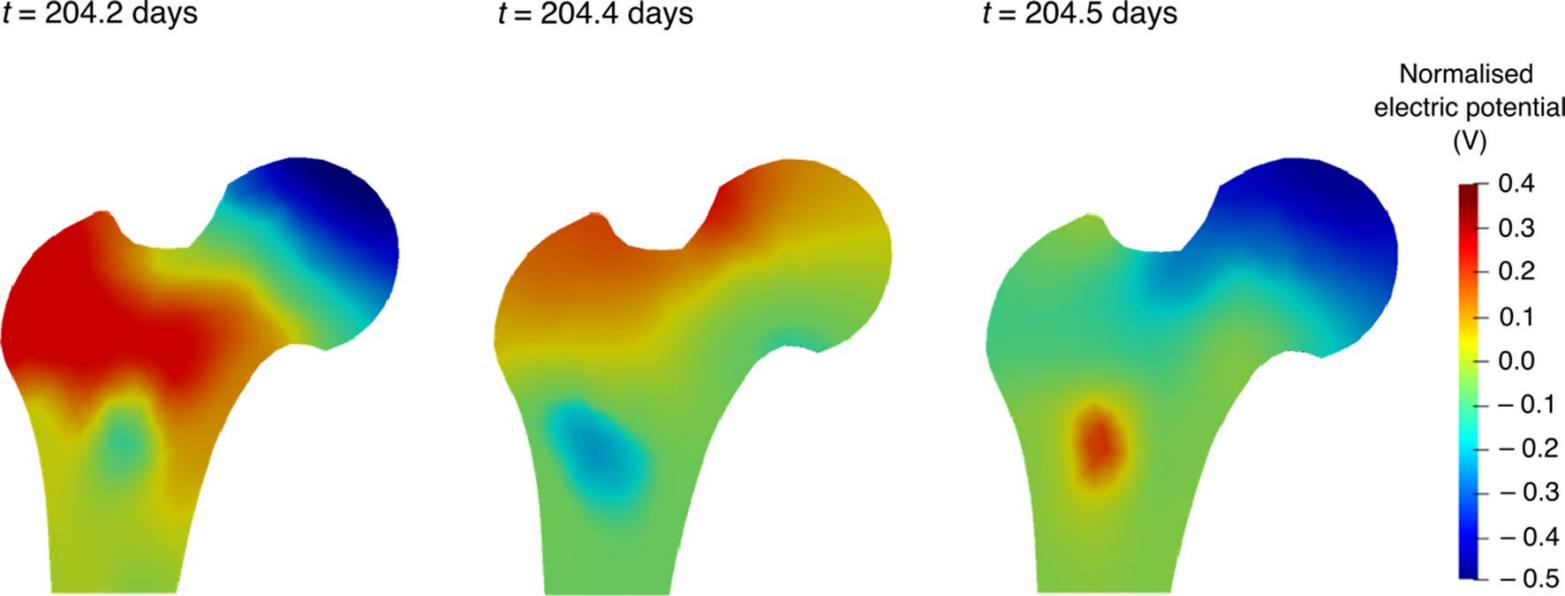
Normalised electric potentials at different time instants *t* of the 204th day

Experimental studies have shown that the applied strain causes changes in the generated electric potential of bone: parts exposed to compressive forces developed negative potentials and parts subjected to tensile forces developed positive potentials (Bassett and Becker 1962; Bassett et al. 1964; Fukada and Yasuda 1957; Zigman et al. 2013). Figure 11 illustrates variations in the distribution of electric potentials and their amplitudes generated during walking due to the application of varying mechanical loads at different time instants of the day (direct piezoelectric effect). Here, the electric potentials generated at different time instants of the day were normalised with respect to their mean amplitude.

For electromechanical simulations, regions with negative potential lead to bone deposition resembling osteoblastic bone formation, whereas regions with positive potential lead to osteoclastic bone resorption (Cerrolaza et al. 2017; Qin and Ye 2004).

### Therapeutic electrical stimulation

**Fig. 12:**
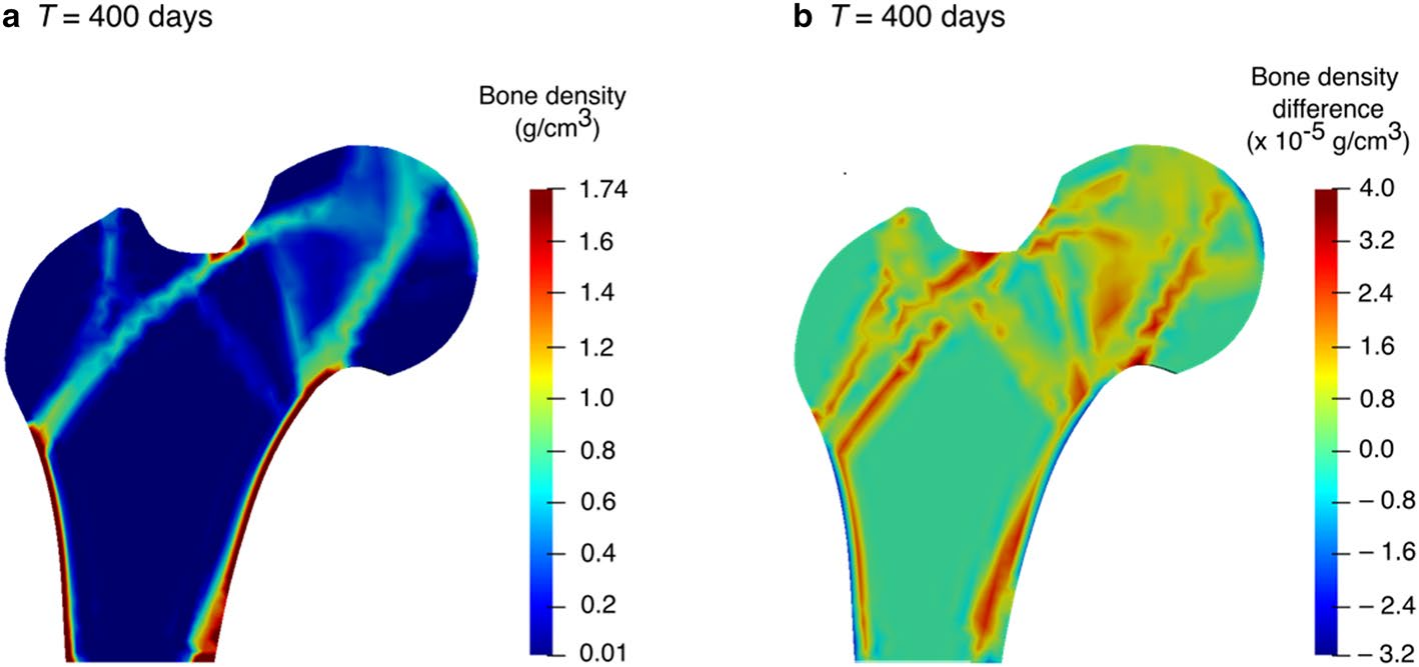
**a** Predicted bone density distribution after a period of reduced physical activity (*T* = 400 days) for piezoelectric strain-adaptive bone remodelling; **b** variations between bone density, after the remodelling period and after the period of reduced physical activity

For a consistent comparison of the simulation results with similar studies from the literature (Fernández et al. 2012a; Cerrolaza et al. 2017), the density distribution obtained after the remodelling period, i.e. at day 300 (Fig. 7b) was considered as an initial state for reduced physical activity simulation and electrical stimulation (see Sect. 2.5). Figure 12a illustrates the density distribution predicted by the piezoelectric strain-adaptive bone remodelling after a period of reduced physical activity, i.e. at *T* = 400 days. To show the regional variations in bone density due to reduced physical activity, the differences between bone density after the remodelling period (300 days) and the period of reduced physical activity (300–400 days) are plotted in Fig. 12b. It is noteworthy that the bone density in trabeculae regions, specifically in the primary compressive trabeculae in the centre of the femoral head was lower than at *T* = 300 days.

**Fig. 13:**
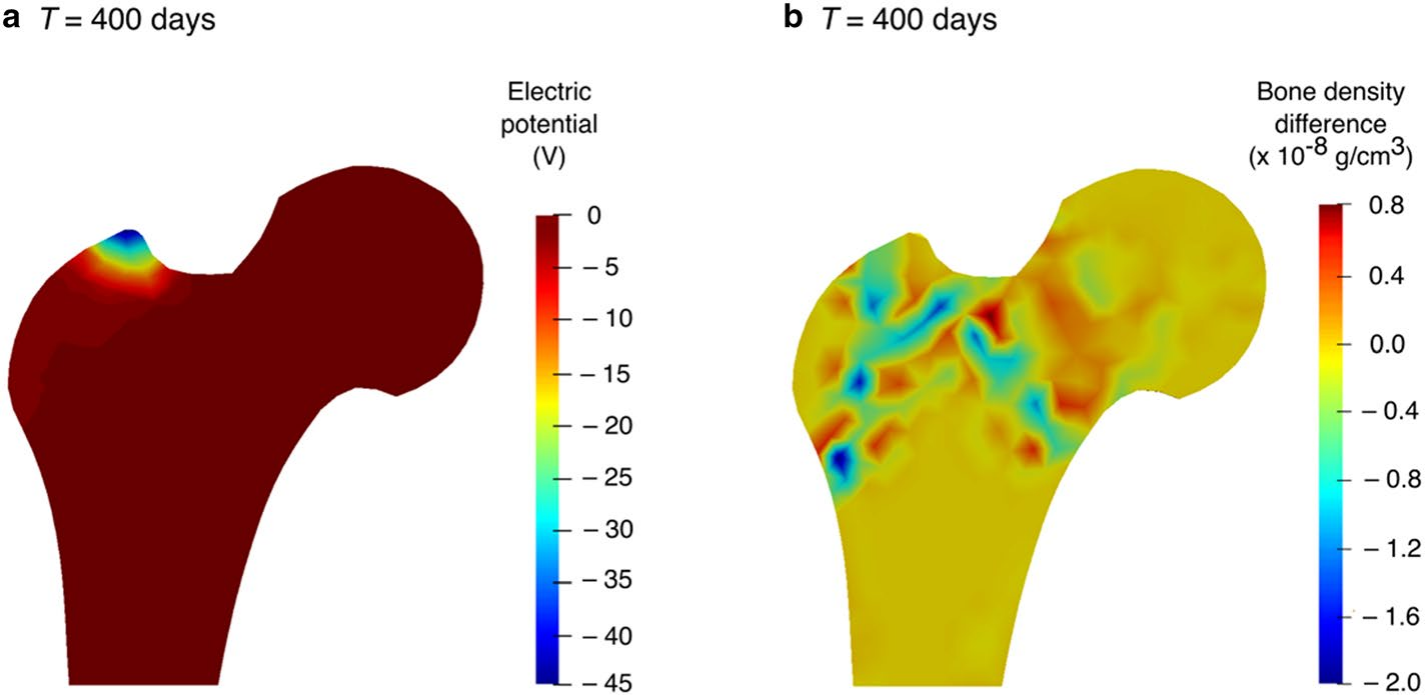
**a** Electric potential generated at time *T* = 400 days, when surface electric charge was applied to the greater trochanter for the period of reduced physical activity; **b** variations between bone density at *T* = 400 days, when therapeutic electrical stimulation was applied and when only mechanical loads were acting

One of the main advantages of taking into account bone piezoelectricity (Sect. 3.3) in computational remodelling studies could be the ability of bone to change its density through therapeutic electrical stimulation (Fernández et al. 2012a). For the surface electric charge applied to the greater trochanter (see Fig. 6b) during the period of reduced physical activity, a negative potential of approximately –45 V was observed (see Fig. 13a). This result compares well with –47 V recently reported by Cerrolaza et al. (2017) using boundary element method (BEM) and –50 V reported by Fernández et al. (2012a) using FEM.

In order to demonstrate the change in bone density resulting from electrical stimulation (inverse piezoelectric effect), the differences between bone density were plotted (Fig. 13b), when an electrical charge was applied in addition to the acting mechanical loads and when only mechanical loads were acting. Although small differences were observed, regions in red represent an increase in the bone density, whereas regions in blue represent a decrease. Compared to the reduction in bone density in trabeculae regions due to reduced physical activity (see Fig. 12b), the therapeutic electrical stimulation mainly affects the region in the vicinity of the point of its application, resulting in a clear increase in bone density in the area between greater trochanter and centre of the femoral head. These results are in line with the findings reported by Fernández et al. (2012a). These preliminary results demonstrated that the coupling between electrical and mechanical loading influences the evolution of bone density distribution. Therefore, therapeutic electrical stimulation could be considered as an additional stimulus to enhance bone remodelling and to reduce bone loss, e.g. due to osteoporosis. The simulation results obtained are in accordance with those reported in (Fernández et al. 2012a) but direct comparison with experimental data is not possible due to the lack of such data in the current literature. Although small changes in bone density after electrical stimulation have been observed in the present study, they were attributed to the matrix piezoelectricity only. These results motivate future development of multi-physics models that allow the modelling of multiple coupled phenomena like matrix piezoelectricity (Fukada and Yasuda 1957), streaming potential (Pollack et al. 1984) and strain generated fluid flow (Cowin et al. 1995) leading to more encouraging results for therapeutic electrical stimulation.

To date, different bone remodelling models have been simulated using commercial software but mostly for the strain-adaptive bone remodelling without considering the piezoelectric effect. Some models have also been implemented using in-house programs developed by individual laboratories and are not publicly available. However, in the presented study, the strain-adaptive bone remodelling models without and with considering the piezoelectric effect were analysed using an open-source framework for the first time and the Python code developed is publicly available on the GitHub repository. To the authors’ knowledge, there is only insufficient information available on the influence of electric stimulation on the bone remodelling process. The piezoelectric strain-adaptive bone remodelling model proposed by Fernández et al. (2012a) is the most recent model that takes the bone piezoelectricity into account and implemented using the FEM. The predicted bone density distributions were evaluated qualitatively by visually comparing with the CT scan and quantitatively by computing the RMS error between simulated BMD and BMD derived from the CT data. By coupling the influences of mechanical loading and electric field on bone response, the direct and inverse piezoelectric effects in bone have been illustrated. The effect of several remodelling parameters such as initial bone density and reference stimulus on the predicted bone density distribution and the piezoelectric and permittivity tensors on the generated electric potentials were also investigated. Additionally, in our present study, the proximal femur geometry derived from the CT image was used instead of the schematic one. This study contributes to a better understanding of the role of bone piezoelectricity in the electro-therapeutic stimulation of bone formation and healing.

Nevertheless, there are certain limitations to the bone remodelling algorithms studied here. These algorithms use the forward Euler method for the time discretisation, which is not very accurate and becomes unstable for large time steps. However, this method has been used here for a fair comparison with previous studies (Fernández et al. 2010, 2012a). Both matrix piezoelectricity and streaming potential are known to contribute significantly to the electromechanical properties of bone (Fernández et al. 2012c) but the coupling between these phenomena is still unavailable. There are large discrepancies in the values of piezoelectric tensor from the literature (Anderson and Eriksson 1970; Fukada 1968; Fotiadis et al. 1999; Qin and Ye 2004; Mohammadkhah et al. 2019). In the context of thermodynamics, the present study is limited by the exclusion of the concepts of energy balance and flow of energy (Holzapfel 2000). Finally, the formation and resorption of bone tissue on the outer bone surface due to external loading (Goda et al. 2016) is not taken into consideration.

As future work, to study the influence of the location and magnitude of an applied electrical stimulus on bone remodelling, it will be applied to the different parts of the human femur. It is well accepted that matrix piezoelectricity in conjunction with streaming potential contributes to the electromechanical properties of bone. Therefore, to better predict the density evolution over time, the constitutive laws for mechanical and electrical behaviour will be modified to incorporate these coupled multi-physics phenomena. To investigate the effect of electrically active implants (Raben et al. 2019; Schmidt et al. 2015; Zimmerman et al.^ 2015^; Potratz et al. 2010) on peri-implant bone remodelling, the piezoelectric bone remodelling algorithm will be simulated for bone implant application using an open-source framework. As another interesting strategy, the reduced activity simulations and electrical stimulation can be performed using the patient-specific bone density distribution as an initial state. The present study will be extended to perform 3D numerical simulations. Furthermore, the boundary conditions can be improved by incorporating results from musculoskeletal multibody simulations (Geier et al. 2019; Kebbach et al. 2020).

## Conclusion

To the authors' best knowledge, this is the first study that has introduced a Python-based open-source software framework to simulate the strain-adaptive bone remodelling in the human femur both without and with piezoelectric effect. The simulation results predicted fairly accurate density distributions that were qualitatively validated by visually comparing with the real CT data and quantitatively validated by computing the RMS error and mean deviation of the absolute differences between the bone apparent density of the simulation and the CT image. The study also demonstrated that at the final time different initial bone density values resulted in different bone density distribution and different average bone density. Furthermore, the bone density distribution results influenced by the piezoelectric effect were compared for normal and reduced physical activity and a reduction in bone density was observed. The therapeutic electrical stimulation in the form of electric charge was applied to the greater trochanter during a period of reduced physical activity and the bone density distribution results were compared with those obtained when only mechanical loads were applied. Our findings demonstrated that the application of an electrical stimulus to the bone surfaces improved bone deposition and these findings are compared with those reported in the literature. This study thus suggests that mechanical loads can be partially replaced by electrical charges that enhance bone density. This study presents an open-source computational approach that contributes to a better understanding of the significance of bone piezoelectricity in the process of remodelling combining the influences of mechanical and electrical loadings on bone response. Therefore, the use of an open-source software framework is very promising for future numerical studies in biomedicine.
